# Studies on the effects of hypothermia combined with hypoxia on rat skeletal muscle and lipid metabolism based on AMPK/PGC1α pathway

**DOI:** 10.1186/s13018-021-02861-0

**Published:** 2021-12-07

**Authors:** Zhang Ruixia, Liu chuanchuan, Guan Lu, Ma Shuang, Zhu Qiang, Tian Xiaofang, Ba Yinggui

**Affiliations:** 1 Endocrinology Department, Qinghai University Affiliated Hospital, Xining, People’s Republic of China; 2 Nephrology Department, Qinghai University Affiliated Hospital, Xining, Qinghai 810001 People’s Republic of China; 3 Key Laboratory of Echinococcosis Research, Qinghai University Affiliated Hospital, Xining, People’s Republic of China; 4 Research Center for High Altitude Medicine, Qinghai University, Xining, People’s Republic of China

**Keywords:** Hypothermia, Hypoxia, AMPK, PGC-1α, Metabolism

## Abstract

**Aim:**

To explore the effects of hypothermia and hypoxia on rat skeletal muscle and lipid metabolism.

**Method:**

Forty male rats were randomly divided into blank group, low-temperature group, hypoxia group, and hypothermia combined with hypoxia group. The body weight of the rats was monitored. The changes of Irisin were detected by ELISA, and LDL, HDL, TC, and TG levels in serum were detected by blood biochemistry. Western blot was used to detect the changes of lipid metabolism-related proteins. CCK8 was used to verify the effect of AMPK/PGC1α on the proliferation of rat skeletal muscle cells.

**Result:**

In the case of cold stimulation and hypoxia, the weight of the rats decreased significantly, and the levels of LDL, HDL, TC, and TG in the serum were abnormal. The activity of fatty acid metabolism factors Irisin, UCP-1, and FABP4 is down-regulated by hypothermia and hypoxia. The activity of fat metabolism-related enzymes, ATGL, HSL, and MGL increased under hypothermia and low oxygen conditions. Hypothermia and hypoxia affected the morphology of skeletal muscle, and AMPK/PGC-1α can regulate the proliferation of skeletal muscle cells.

**Conclusion:**

Hypothermia and hypoxia can reduce the body weight of rats, and affect the structure of skeletal muscle to promote lipid metabolism through AMPK/PGC-1α signaling pathway.

## Introduction

With the development of tourism, more and more domestic and foreign tourists go to Qinghai Province, China, for vacation, which belongs to a plateau continental climate with low atmospheric pressure, low oxygen partial pressure, and low temperature. However, people remaining in the plateau environment for a long time will cause weight loss [1, 2]. The relationship between hypothermia combined with hypoxia exposure and weight loss is not clear, which may be related to hypoxia by increasing the body's metabolism and reducing appetite. Low oxygen and hypothermia may be the primary environmental factors that affect human health and work capacity in plateau areas.

Skeletal muscle is one of the energy-consuming tissues, which is also an important place for the energy metabolism of the body [3]. Its metabolic homeostasis is the basic premise and important guarantee for maintaining the health of skeletal muscle and even the health of the entire body [4]. AMPK (Adenosine 5’-monophosphate (AMP)-activated protein kinase) is an energy sensor of skeletal muscle, and PGC-1α (Peroxisome proliferator-activated receptor gamma coactivator-1 alpha) plays an important role in skeletal muscle fatty acid oxidation and metabolism [5, 6]. Previous studies have confirmed that under normoxia, AMPK as an important regulator of PGC-1α, can activate PGC-1α and strengthen skeletal muscle [7, 8]. UCP-1 (Uncoupling protein 1) appears in mitochondria to promote fatty acid oxidation. Irisin can increase the expression of UCP-1 in adipocytes and also can promote the differentiation of osteoblasts in vitro [9]. FABP4 (Fatty acid-binding protein 4) are small proteins of 14–15 KD abundantly expressed in cells, which can regulate lipid transport, energy storage [10]. At the same time, the proteins ATGL (Adipose triglyceride lipase), HSL (Hormone-sensitive lipase), and MGL (Recombinant Lipase, Monoacylglycerol) are closely related to lipid metabolism, which all participate in fat metabolism [11]. However, the effects of AMPK/PGC1-α on skeletal muscle and fat metabolism under hypothermia and low oxygen environment and its underlying mechanism are still unclear.

In this study, we discovered the expression of AMPK in rats, examined its influence on the proliferation of skeletal muscle myoblasts L6, and recorded the effects of hypothermia and hypoxia on rat body weight and skeletal muscle pathology, as well as irisin and LDL, HDL, TC, TG levels in serum I, and explored the potential mechanisms involved in energy metabolism.

## Materials and methods

### Animals and grouping

Forty SD rats (140–160 g) were purchased from Shanghai Jiesjie Experimental Animal Co., Ltd. (Animal Certificate No. 2010002604739). Animals were reared in a 12 h/12 h light–dark alternate environment, and they had free access to water. The animal experiment was approved by the Ethics Committee of the Affiliated Hospital of Qinghai University and complied with the regulations of China's "Guidelines for the Feeding, Management and Use of Laboratory Animals".

The animals are randomly divided into four groups. (A) Blank group: Rats reared in normal oxygen concentration at a temperature of 22 ± 2℃. (B) Low-temperature group: Rats are reared in the environment with an oxygen concentration of 21% and temperature (4–6℃) for 28 days. (C) Hypoxia group: Rats are placed in a continuous low-pressure oxygen chamber, which simulates a 5000 m plateau environment with an oxygen concentration of about 10% and a temperature of 22 ± 2 °C for 28 days. (D) Hypoxic hypothermia: Rats are placed in a low-pressure hypoxic chamber with an oxygen concentration of about 10% and a refrigerator temperature of 4–6 °C for 28 days. All experimental rats were fed with experimental rat pellets, and they had free access to food and water.

### ELISA assay

The supernatant of blood was added to the Irisin ELISA plate (xl-Eh0436, XLPCC) and incubated at 25℃ for 2 h. After washing the plate with PBS 5 times, the enzyme-linked affinity was added to the plate and incubated at 25 ℃ for 2 h. Then, the plate was washed with PBS. After that, the substrate solution was added to the plate, incubated at 25℃, and protected from light. Finally, the reaction terminated by adding the termination solution. A gradient concentration of standard irisin was prepared to make a standard curve. The absorbance was measured at 450 nm, and the concentration of Irisin in the sample was calculated (C).

### Blood biochemistry analysis

Venous blood was collected from rats and left to stand for 30 min, then centrifuged at 3 000 r·min^−1^ for 15 min, and the supernatant was collected and stored in a refrigerator at − 80 ℃. The serum levels of TC, TG, LDL-C, and HDL-C in each group of rats were quantified by a biochemical method using a Hitachi 7600 automatic biochemical analyzer.

### Histological examination of skeletal muscle

Skeletal muscle was taken from mice under ice bath immediately after execution, fixed in 10% formalin solution, and pathological sections were routinely paraffin-embedded, sectioned, and HE-stained (Roche Applied Science, Germany) to observe the degeneration and necrosis of skeletal muscle cells and the pathological changes of fiber proliferation. Images were captured with a microscope (Olympus Corporation, Japan).

### Western blot assay

Western blot was used to detect the expression of proliferation and metastasis-related proteins. The cells and tissue were digested and collected to lysis with ultrasound for 5 min. The protein concentration was determined by the Coomassie brilliant blue method. Polyacrylamide gel electrophoresis was performed with 50 μg protein mixture in each electrophoresis hole. Then the protein was transferred to the membrane. Adding the primary and secondary antibodies AMPK (cell signaling), p-AMPK (santacruze), PGC1α (ABCAM), UCP-1 (ABCAM), FABP4 (BioVision), ATGL (FineTest), HSL (ABCAM), MGL (ABCAM), respectively, for hybridization. ECL reaction, darkroom development, exposure. Bio-rad software analyzed the ratio of the protein content of each electrophoresis band of the protein content of β-actin.

### Cell culture

Skeletal muscle myoblast cells of rat L6 were purchased from Procell Life Science & Technology Co., Ltd. (Wuhan, China). L6 is cultured with DMEM medium containing 10% FBS. When the cells grow to cover about 80–90% of the bottom surface of the culture flask, discard the culture fluid in the culture flask, add 3 mL PBS, wash gently, and discard PBS solution, add 2 mL trypsin digestion solution to digest for 1 min, discard the trypsin digestion solution, 2 mL 1640 medium to elute the cells, take 20% for passage, and place it in a 37 ℃, 5% CO_2_ cell incubator.

### CCK-8 assay

Cells were seeded into 96-well plates and cultured overnight, and the medium was replaced after 12 h, with 5 repeating holes set in each group. Absorbance (A) at 450 nm at the appointed time points was recorded in accordance with the Cell Counting Kit-8 (CCK-8; Dojindo Laboratories, Kumamoto, Japan). Finally, the statistics graph of growth inhibition rate was drawn according to the cell proliferation. The concentration of the inhibitor AMPK is 1 μmol·L^−1^. The equations used were as follows: (1 − OD of observation group/OD of blank group) * 100.

### Statistical analysis

The data obtained in the experiment are all statistically analyzed by GraphPad Prism 8.0 software. One-way ANOVA with Dunnett’s multiple comparisons among more than three groups expressed as *x* ± *s*, *P* < 0. 05 means the difference is statistically significant.

## Results

### Effects of hypothermia and hypoxia on lipid metabolism in rats

**Fig. 1:**
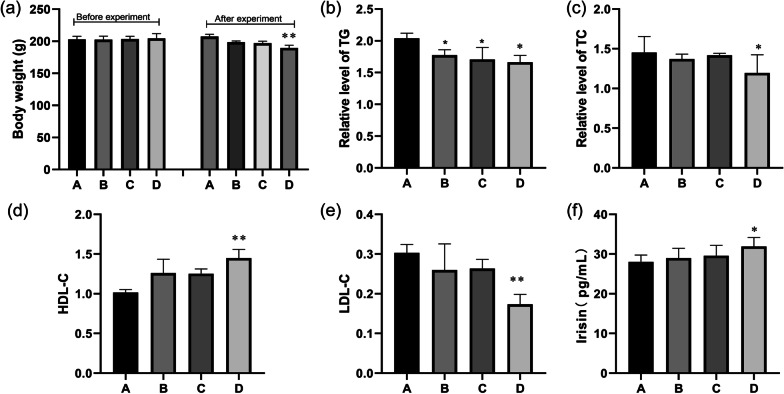
Effects of hypoxia and hypothermia on factors related to body weight and lipid metabolism in rats. **a** Weight changes of rats. **b** TG in each group. **c** TC in each group. **d** HDL-C in each group. **e** LDL-C in each group. **f** Irisin in each group. A: Blank group. B: Hypoxic group. C: Hypothermic group. D: Hypoxic hypothermic group. The results were presented as the mean ± standard deviation. *P < 0.05 and **P < 0.01, ***P < 0.001 compared with the blank group (A)

To verify the effect of hypothermia and hypoxia on lipid metabolism in rats, the bodyweight of rats was recorded, as well as LDL, HDL, TC, TG, and Irisin in serum. There was no significant difference in the bodyweight of rats before the experiment. After 4 weeks of intervention, the bodyweight of B (hypoxic group) and C (hypothermic group) were both smaller than A (blank group), but no significant difference (*P* > 0.05), and the bodyweight of D (hypoxic hypothermic group) decreased more significantly compared with A (*P* < 0.05), Fig. 1a. As shown in Fig. 1b, c and e, the levels of TG, TC, and LDL-C in the experimental intervention group (B, C, D) were significantly lower than those in the blank group (A). The levels of HDL-C and Irisin in the experimental intervention groups were higher than those in the black group. Compared with those in the A group, the levels of the abovementioned factors related to lipid metabolism were significantly increased in the D group (*P* < 0.05), figure (d and f).

### Histological effects of hypothermia and hypoxia on skeletal muscle of rats

**Fig. 2:**
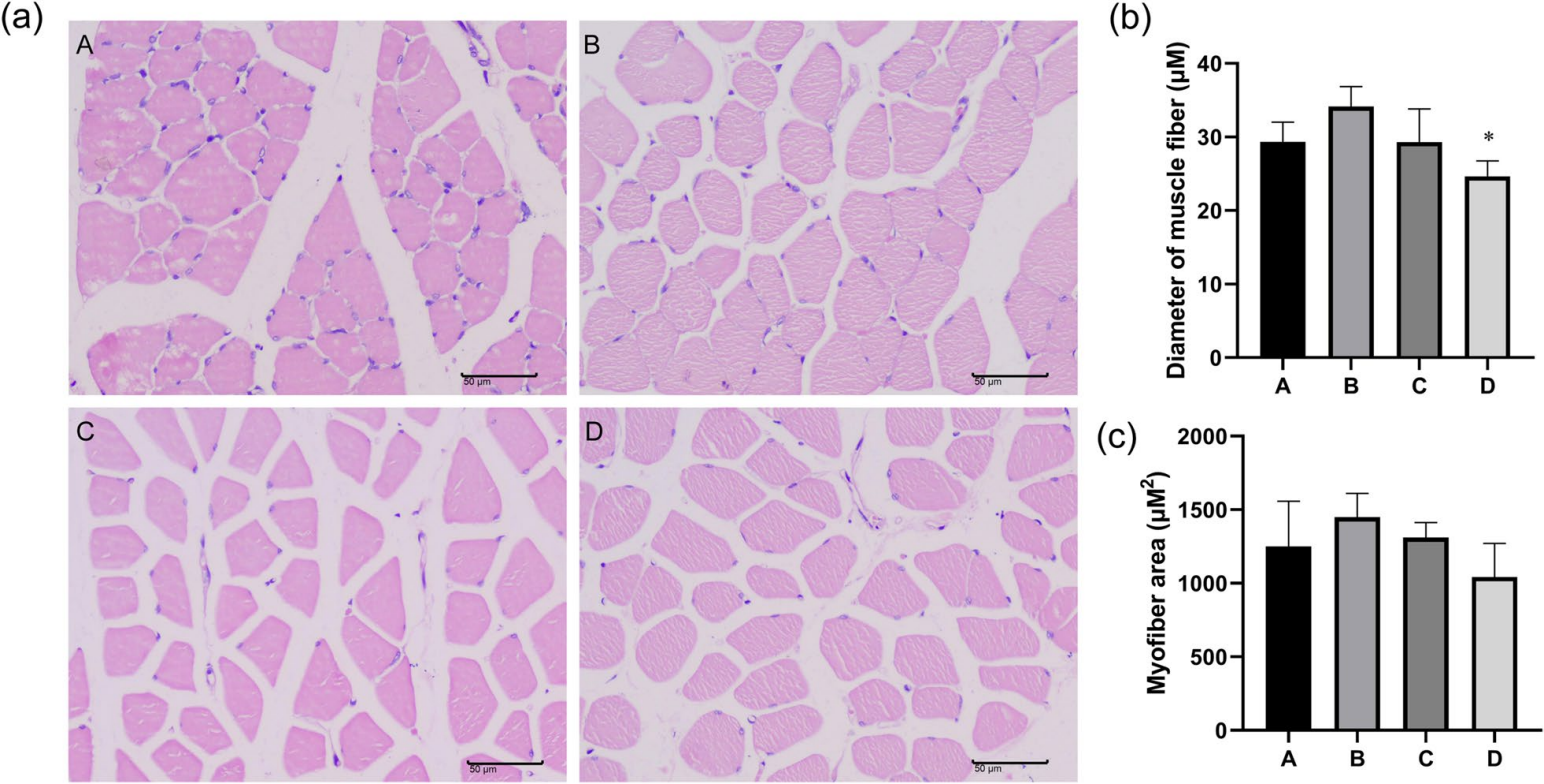
Histological effects of hypothermia and hypoxia on skeletal muscle of rats. **a** Images of skeletal muscle tissue following hematoxylin–eosin (H&E) staining. **b** Statistics result of muscle fiber diameter. **c** Statistics result of muscle fiber area. A: Blank group. B: Hypoxic group. C: Hypothermic group. D: Hypoxic hypothermic group. The results were presented as the mean ± standard deviation. *P < 0.05 and **P < 0.01, ***P < 0.001 compared with the blank group (A)

Pathological changes in the skeletal muscle tissue were measured with HE staining (Fig. 2a–c). Compared with the A groups, the experimental intervention group (B, C, and D) lead to muscle fibers get thinner. Hypothermia and anoxic had no effects on inflammatory cell infiltration and pathological change in each group. However, the cell spaces of skeletal muscle tissue become larger, and the area of muscle fibers becomes larger and the number of muscle fibers increases.

### The effect of hypoxia and hypothermia on the lipid metabolism-related proteins of rats through the AMPK/PGC1α pathway

**Fig. 3:**
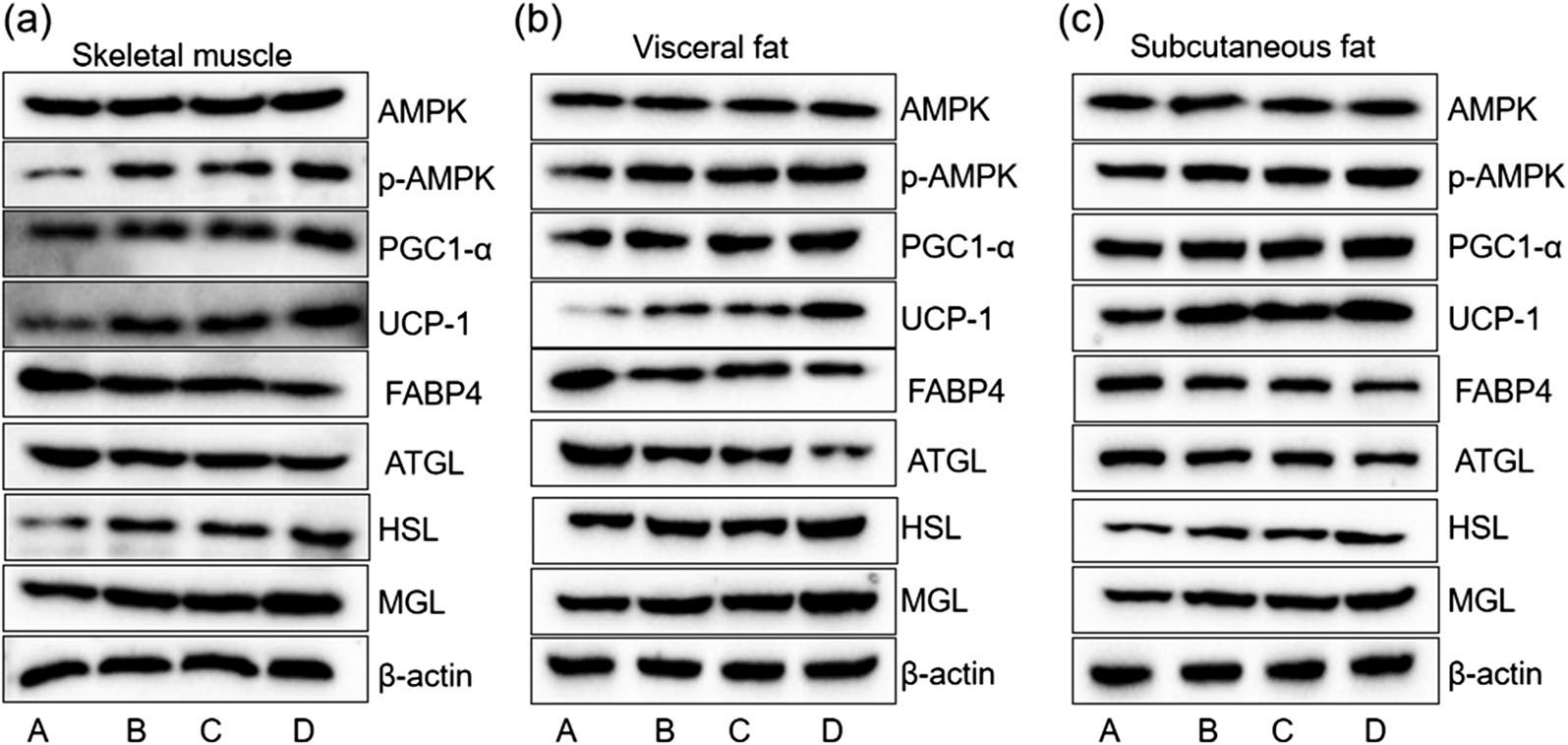
AMPK, p-AMPK, PGC1α, UCP-1, FABP4, ATGL, HSL, and MGL protein levels in skeletal muscle, subcutaneous fat, and visceral fat of rats. **a** The protein of skeletal muscle tissue. **b** The protein of visceral fat. **c** The protein of skeletal subcutaneous fat. A: Blank group. B: Hypoxic group. C: Hypothermic group. D: Hypoxic and hypothermic group

AMPK/PGC1α pathway has been known to be the energy perception of skeletal muscle and plays an important role in fatty acid oxidation metabolism. To further investigate the mechanisms by which AMPK/PGC1α activates the lipid metabolism, including brown fat marker UCP-1 as described above, and fatty acid metabolism-related enzymes ATGL, HSL, MGL were detected the effect of low oxygen and hypothermia. As shown in Fig. 3a–c, hypothermia and low oxygen resulted in an obvious increase in the production of phosphorylated AMPK and PGC1α, and Simultaneous treatment of hypothermia and low oxygen has more obvious effect. The expression of UCP-1 significantly increased in the experimental intervention group (B, C, and D) compared with group A, which suggested an increase in brown fat. Moreover, HSL upregulated, ATGC, and FABP4 downregulated due to hypothermia with hypoxia, which indicated accelerated metabolism of fatty acid.

### Effects of hypoxia and hypothermia on the proliferative activity of rat skeletal muscle myogenic cells

**Fig. 4:**
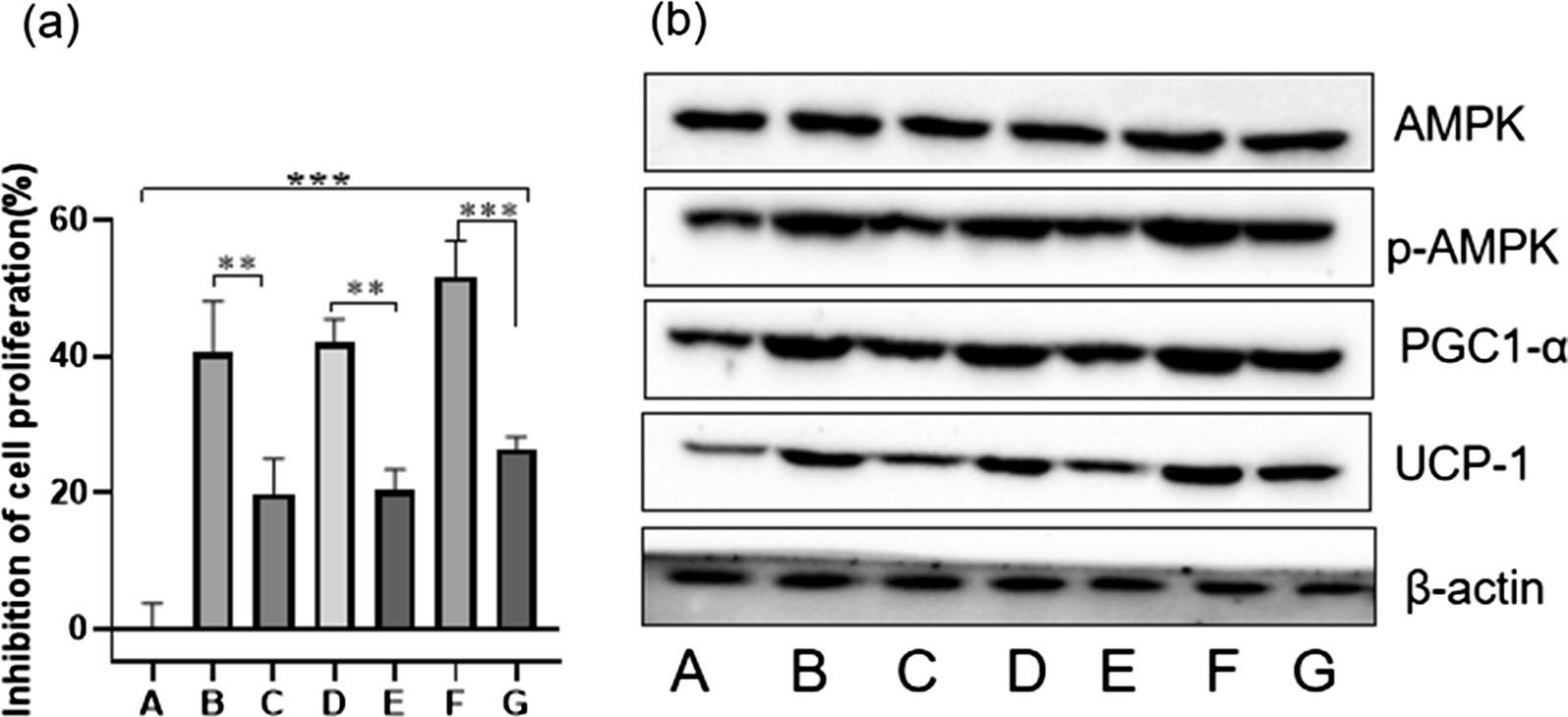
Effects of AMPK on the proliferation activity of rat skeletal muscle myoblasts. **a** Inhibition rate of cell proliferation. **b** The protein of skeletal muscle cells L6. A: Blank group. B: Hypoxic group. C: Hypoxic with AMPK inhibitor group. D: Hypothermic group. E: Hypothermic with AMPK inhibitor group. F: Hypoxic hypothermic group. G: Hypoxic hypothermic with AMPK inhibitor group. The results were presented as the mean ± standard deviation. *P < 0.05 and **P < 0.01, ***P < 0.001 compared with the blank group (A)

To further investigate the effects of hypothermia and hypoxia on skeletal muscle through activation of the AMPK/PGC1α pathway, L6 cells were treated with low-temperature hypoxia and AMPK inhibitor dorsomorphin (Compound C), detected by CCK8 and western blot. Compared with the A (blank) group, B (hypothermia) group, D (hypoxia) group, F (hypothermia and hypoxia) group has a higher growth inhibition rate. Compound C can reduce the high rate caused by hypothermia and hypoxia (*P* < 0.05), which suggested that the growth of skeletal muscle cells is related to the AMPK pathway (Fig. 4a). To further testify the signal transduction pathway of AMPK, a western blot assay was performed to detect the expression of AMPK/PGC1α in L6 cells (Fig. 4b). Compared with the A (blank) group, hypothermia and hypoxia can significantly overexpress p-AMPK, PGC1α, and UCP-1. Compound C can inhibit the expression of these proteins. These data indicated that the activation of p-AMPK can promote the expression of PGC1α and UCP-1.

## Discussion

Due to the tremendous environmental differences between high altitude and inland plains, the health problems of people in hypothermia and low oxygen environments have become increasingly prominent [12]. Many studies have pointed out that residents living in high-altitude areas may suffer from metabolic syndrome and vascular dysfunction [13]. This experiment confirmed that hypothermia and hypoxia significantly enhance AMPK phosphorylation and up-regulate the expression of PGC-1α protein, which may be one of its mechanisms to improve the lipid metabolism disorder of rat skeletal muscle cells.

Exposure of body tissues or cells to hypoxic conditions often causes physiological or pathological changes in the body and even changes in gene levels [14]. The body often has strong adaptability to the corresponding change in the body's physiology or morphological structure to adapt to the changes in environmental stimuli [15]. Studies have shown that under the stimulation of low-oxygen environmental exposure, the intake of fatty acids and lipid storage of body cells will be reduced [16]. Studies have found that long-term low-oxygen exposure stimulation can enhance the body’s fatty acid oxidation metabolism [17]. This article uses rats as a model animal, which will provide a theoretical basis for the study of such phenomena in humans.

In 2012, Irisin was first identified as one of the myokines and an adipokine closely associated with obesity and related metabolic diseases [18]. Irisin is secreted by skeletal muscle hydrolyzed and cleaved by fibronectin type III domain-containing protein 5 (FNDC5) by proteolytic enzymes and released into the blood. Irisin regulated by PGC-1α, can promote white fat convert to brown fat, significantly reducing body mass and insulin resistance induced by the high-fat diet [19]. It is clear that hypothermia and low oxygen significantly caused weight loss in the experimental rats, which are similar to Jacopo's study [13] that environmental factors at high altitude caused weight loss. Studies have shown that aerobic hypothermia and low oxygen can affect the metabolism of blood lipids, as well as affect various indicators related to lipid metabolism. Hypoxic and hypothermia improve serum lipids in patients with hyperlipidemia by lowering serum triglyceride (TG) levels, total cholesterol (TC), and low-density lipoprotein cholesterol (LDL-C) levels while increasing high-density lipoprotein cholesterol (HDL-C) levels.

Rats are raised in a low temperature and low oxygen environment, which restores the natural environment of Xining City, Qinghai Province, and provides more reliable experimental conditions for the treatment of skeletal muscle and lipid metabolism in this area. Low temperature and hypoxia can upregulate UCP-1 to achieve more heat production, and UCP-1 can transport protons across the inner mitochondrial membrane, but this process destroys oxidative phosphorylation and causes more than 50% of the energy lost in the form of heat, and the body’s demand for ATP forces it to consume more energy to synthesize ATP, thereby promoting fat catabolism [20]. The down-regulation of FABP4 under hypothermia and hypoxia can reduce the reversible combination with saturated and unsaturated long-chain fatty acids and other hydrophobic ligands, and effectively reduce the plasma triglyceride and cholesterol levels in mice, and improve lipid metabolism [21]. The lipolysis process is catalyzed by three different enzymes. In the beginning, the triglycerides was catalyzed by ATGL through removing one molecule of fatty acids to obtain diacylglycerols (DAG). Then, the conversion of DAG to monoacylglycerols (MAG) was through HSL (Hormone-Sensitive Lipase) by removing one molecule of fatty acids. Finally, MGL (Monoglyceride Lipase) completes the catalysis of monoacylglycerol, and the final product is one molecule of fatty acid and one molecule of glycerol [21].

Concerning H&E and western blot experiments, it can be found that hypothermia and hypoxia can change the structure of rat skeletal muscle cells, enhance AMPK phosphorylation, up-regulate the expression of PGC-1α, and regulate the lipid metabolism of rat skeletal muscle cells. AMPK is a powerful potential target for metabolic diseases and energy metabolism disorders. Studies have found that hypoxia or exercise can promote the up-regulation of AMPK expression [5, 22]. As an important upstream regulator of PGC-1α, AMPK can directly promote the expression of PGC-1α, thereby enhancing skeletal muscle mitochondria and promoting fatty acid oxidation [7]. In order to further verify the effect of AMPK/PGC-1α on skeletal muscle cells under hypothermia and low oxygen conditions, AMPK inhibitor compound C was applied to the CCK8 assay. Hypothermia and low oxygen can inhibit the cell proliferation of L6, and compound C can significantly reverse this reaction. Western blot indicated that compound C can down-regulate AMPK phosphorylation and up-regulation of PGC-1α caused by hypothermia and hypoxia. Therefore, it indicates that the AMPK/PGC-1α signaling pathway can affect the biological behavior of L6, and further reveals that AMPK/PGC-1α may affect the skeletal muscle of rats and affect metabolism.

## Conclusion

In this study, the effect of hypothermia and hypoxia on skeletal muscle AMPK/PGC-1α and its downstream molecules in regulating skeletal muscle lipid metabolism was demonstrated, which may be of its mechanisms to promote lipid metabolism in rat skeletal muscle cells. There is no relevant research at present. However, only AMPK inhibitor was used in this experiment. This article does not delve into the mechanisms involved, and the proteins downstream of AMPK should be studied one by one. It is necessary to carry out gene knockout and RNA interference to prove the role of the AMPK/PGC-1α signaling pathway in the effects of hypothermia and hypoxia. In the next step, gene knockout rats can be selected for in-depth research.

## Data Availability

Not applicable.

## Abbreviations

LDL: Low density lipoprotein
HDL: High-density lipoprotein
TC: Serum total cholesterol
TG: Triglyceride
UCP-1: Uncoupling protein 1
FABP4: Fatty acid-binding protein 4
ATGL: Adipose triglyceride lipase
HSL: Hormone-sensitive lipase
MGL: Recombinant lipase
